# A Spectrum-Saving Transmission Method in Multi-Antenna Satellite Communication Star Networks: Sharing the Frequency with Terminals

**DOI:** 10.3390/e25010113

**Published:** 2023-01-05

**Authors:** Tian Li, Xuekun Hao, Xinwei Yue

**Affiliations:** 1 Science and Technology on Communication Networks Laboratory, The 54th Research Institute of China Electronics Technology Group Corporation, Shijiazhuang 050081, China; 2 Shanghai Satellite Network Corporation Limited, Shanghai 201210, China; 3 School of Information and Communication Engineering, Beijing Information Science and Technology University, Beijing 100101, China

**Keywords:** multi-antenna satellite, spectrum-saving transmission, star network, ergodic sum rate

## Abstract

Satellite communication networks have gradually been recognized as an effective way to enhance the ground-based wireless communication. Considering the weight restriction of payloads, multi-antenna technologies have recently come into use on satellite platforms, and are capable of generating beams flexibly to provide services. To avoid incurring interferences, adjacent beams are designed to take different spectral resources. Unfortunately, this may limit the simultaneously accessed terminals since the spectrum cannot be fully used. In this paper, we propose a spectrum-saving transmission method in a satellite star network, where terminals communicate with each other through the hub station. Taking advantage of the great transmission capability differences of the hub station and terminals, we could allocate them the same spectral resources. Specifically, it is not necessary to use exclusive frequency bands for terminals.The proposed method can play a significant role when large numbers of users need to access the system with limited spectrum resource. To give a deep insight into the spectrum-saving method, the expressions of ergodic sum-rate are provided, and the impact of the number of accessed terminals is further analyzed. Simulation results validate the advantage of the proposed method in terms of bit error rate and ergodic sum rate.

## 1. Introduction

As a significant part of space information networks, satellite communication (SATCOM) has been widely applied to provide global wireless links [1]. Motivated by the seamless coverage character of SATCOM, researchers began to study how the SATCOM helps the terrestrial wireless networks, especially the 5G and beyond [2,3,4,5]. As one of the significant achievements, the nonterrestrial network (NTN) was proposed in Release 16 and formally defined in Release 17 by 3GPP [6]. In NTN, one or more satellites work as relays which can help exchange data among user terminals (UTs). Meanwhile, gateways are required to manage the NTN, and can connect the NTN with terrestrial networks [7,8]. Due to the severe signal attenuation of space-to-ground links, gateways are often equipped with high transmit–receive gain antennas, which have the ability to amplify UTs’ signals in SATCOM star networks [9,10,11].

A typical SATCOM star network consists of a gateway, called hub station (HS) in this scenario, and multiple UTs. Two UTs could communicate with each other in a UT-to-HS-to-UT two-hop-wise manner, where the HS works in the amply-and-forward mode. As another usage of the star network, the HS broadcasts to UTs and collects information in the backward link. This mechanism has been widely applied in multimedia and Internet of Things [12,13]. To meet the transmission requirements of extensive UTs, multiple access (MA) technologies have been studied. The authors in [14] provided possible application scenarios of SATCOM star networks. In addition, commonly used MA techniques, such as time division multiplexing and time division multiple access (TDM/TDMA) and frequency division multiple access (FDMA), are also investigated. In order to give instructions for MA techniques in the SATCOM star network, the authors in [15] studied measurement methods in terms of the transmitting performance. By building a ground-based measurement system with a UT, ref. [15] gave testing results of TDMA and multi-frequency TDMA (MF-TDMA) on the aspects of power consumption and spectrum envelope. Motivated by the advanced MA schemes in terrestrial wireless networks, high-spectral-efficiency MA solutions such as nonorthogonal multiple access (NOMA) were developed. The authors in [16] gave deep insights into the terrestrial–satellite integrated network, where the SATCOM works as a supplemental means for the terrestrial NOMA network. Users with poor channel conditions would be served by the satellite. To maximize the channel capacity, ref. [16] further proposed the user-pairing strategy, and the power allocation scheme was also studied. Realizing that the SATCOM would be fully integrated in ground-based wireless network in the future, the same authors discussed a terrestrial–satellite jointly beamforming method in their work [17]. Specially, optimal beamforming vectors were derived to improve the channel capacity. In [18], the authors proposed a two-phase NOMA in two-user SATCOM networks. The co-channel users decode NOMA signals in the first phase, where the strong user could obtain the weak user’s signal as well. Then, the strong user pushes forward useful signals to the weak user to enhance the signal-to-noise ratio (SNR). For the sake of analyzing how the NOMA behaves in a shadow fading environment, the authors in [19] investigated multi-user NOMA over shadowed Rician fading channels. The outage probability was derived to validate the advancement compared with conventional MA schemes. Inspired by NOMA, we developed a co-carrier transmission method in our previous work [20]. Different from the works in the above, the co-carrier method occupies partial bandwidth instead of the whole spectral resource acquired in conventional NOMA. Particularly, ref. [20] further gave instructions on pairing suitable users for co-carrier transmission.

Recognizing the benefits of the multi-antenna technique, researchers launched the development of multiple antennas in SATCOM. Due to the poor scattering character and limited processing capability on satellites, a multi-antenna with phase controller is usually considered in SATCOM [21,22,23]. For the coexistence of service and backhaul links in a satellite–terrestrial integrated network, the authors in [22] designed analog beamforming vectors to mitigate the co-channel interference. To flexibly meet the various requirements of different regions, the authors in [23] proposed a novel amplitude controllable phased array antenna. Precisely, the phase and amplitude can be adjusted separately for each beam. To the best of our knowledge, phased array antennas have not been fully discussed in SATCOM star networks, especially in spectral-resource-limited scenarios.

In this paper, we propose a spectrum-saving transmission method in the SATCOM star network, where the satellite and UTs are equipped with multiple antennas. Realizing that the antenna gain at the hub station is much higher than that at UTs, we allocate the UTs within the spectral resource occupied by the HS. To distinguish the required information from the mixed signals, successive interference cancellation (SIC) and parallel filtering is designed at the HS. The ergodic sum rate is further derived for performance evaluation. In addition, the impact of the number of accessed UTs is also analyzed theoretically. Simulation results are shown from the aspects of ergodic sum rate and bit error rate (BER).

The main contributions of the paper can be summarized as follows:

- By fully analyzing the characters of the SATCOM star network, we found the transmit and receive gain differences of the antennas equipped at the HS and UTs, which could be equivalent to the channel gain difference for the receiver.
- We proposed a spectrum-saving transmission method where the HS could share the bandwidth with UTs. Thanks to the novel scheme, we do not need to allocate an exclusive frequency band for the UTs, which may spare plenty of spectral resource.
- To facilitate performance evaluation, the expression of ergodic sum rates for the proposed and conventional methods were derived. Furthermore, system availability was also defined to analyze the impact of the accessed UTs, which could help to design a more efficient star network.

The rest of the paper is organized as follows. Section 2 describes the system model of a SATCOM star network, and presents the conventional spectrum isolation transmission method. In Section 3, the spectrum-saving scheme is proposed, where the HS shares the frequency band with UTs. To evaluate the performance, ergodic sum rate is derived and analyzed theoretically in Section 4. The impact of UTs on the novel transmission method is also studied. Simulation results of the ergodic sum rate and BER are presented and discussed in Section 5, and the paper is concluded in Section 6.

Notation: Vectors and matrices are presented by lowercase and uppercase bold letters, respectively. The transpose and Hermitian transpose are denoted by superscripts T and H, respectively. The statistical expectation and the probability are presented by E[·] and P(·), respectively, while CN(a,R) denotes the distribution of circularly symmetric complex Gaussian (CSCG) random vectors with mean vector a and covariance matrix R.

## 2. System Model

### 2.1. Description of the SATCOM Star Network

**Figure 1:**
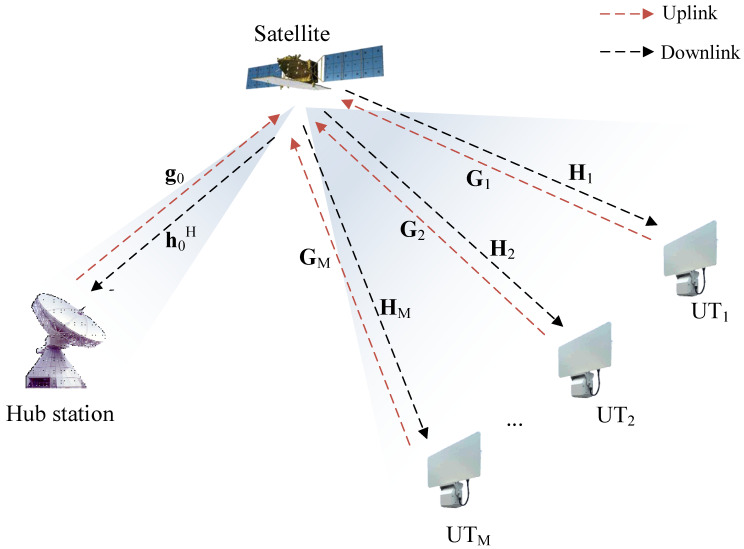
The SATCOM star network.

The SATCOM star network considered in this study is illustrated in Figure 1. The HS broadcasts to the *M* UTs and collects information from the UTs in backward links. Different from the full-duplex in [24], we apply frequency division duplexing in the uplink and downlink which could help to avoid self-interference. Specifically, the system works in transparent mode, which indicates that the satellite amplifies signals without digital processing [25]. Considering the mobility of the UTs, we assume that uniform planar arrays (UPAs) are deployed at the satellite and UTs while the HS is equipped with a reflector antenna (since the HS appears as a fixed satellite station which does not have constraints on antenna weight, we consider that a reflector antenna is equipped at the HS). In the star network, the satellite generates two beams (called feeder beam and service beam) which could cover the HS and UTs separately.

For notational convenience, yz-plane (note that the results of the paper could still hold for other orientation UPAs) UPAs with $N_{y}^{S}\times N_{z}^{S}$ and $N_{y}^{U}\times N_{z}^{U}$ antenna elements are employed at the satellite and UTs. Let ϕ (θ) and ω (υ) denote the azimuth (elevation) angles of arrival at the satellite and UTs, respectively. The azimuth (elevation) angles of departure are denoted by $\phi^{{}^{\prime }}$ ($\theta^{{}^{\prime }}$) and $\omega^{{}^{\prime }}$ ($\upsilon^{{}^{\prime }}$), correspondingly. Then, the receive array response vectors at the satellite and UTs can be derived as

(1) $$\begin{array}{c} a\left(\phi ,\theta \right)=\frac{1}{\sqrt{N_{y}^{S}N_{z}^{S}}}{\left[1,\ldots ,e^{jk\mu (m\sin(\phi )\sin(\theta )+n\cos(\theta ))},\ldots ,e^{jk\mu (\left(N_{y}^{S}-1\right)\sin\left(\phi \right)\sin\left(\theta \right)+\left(N_{z}^{S}-1\right)\cos\left(\theta \right))}\right]}^{T} \end{array}$$

and

(2) $$\begin{array}{c} b\left(\omega ,\upsilon \right)=\frac{1}{\sqrt{N_{y}^{U}N_{z}^{U}}}{\left[1,\ldots ,e^{jk\mu (m\sin(\omega )\sin(\upsilon )+n\cos(\upsilon ))},\ldots ,e^{jk\mu (\left(N_{y}^{U}-1\right)\sin\left(\omega \right)\sin\left(\upsilon \right)+\left(N_{z}^{U}-1\right)\cos\left(\upsilon \right))}\right]}^{T}, \end{array}$$

where k=2π/λ, and μ=λ/2 denotes the interelement spacing. Here, λ is the carrier wavelength. The transmit array response vectors can be denoted as $a(\phi^{{}^{\prime }},\theta^{{}^{\prime }})$ and $b(\omega^{{}^{\prime }},\upsilon^{{}^{\prime }})$, correspondingly. We denote $G_{t,0}$ and $G_{r,0}$ as transmit and receive antenna gains at the HS, respectively. The normal-direction gains of transmit (receive) beams at the satellite and UT UPAs are represented by $E_{t}$ ($E_{r}$) and $G_{t}$ ($G_{r}$), respectively. For the reason of the long travel distance of space-to-ground links, signals may mainly experience large-scale fading, while small-scale fading only exists in the downlink. Let $l\left(d\right)=\sqrt{{\left(\lambda /\left(4\pi d\right)\right)}^{2}}$ denote the large-scale channel coefficient, where *d* is the orbit height. The uplink and downlink channels between the HS and satellite can be modeled as

(3) $$\begin{array}{c} g_{0}=a\left(\phi_{0},\theta_{0}\right)\sqrt{E_{r}}l\left(d\right)\sqrt{G_{t,0}} \end{array}$$

and

(4) $$\begin{array}{c} h_{0}^{H}=\rho_{0}\sqrt{G_{r,0}}l\left(d\right)\sqrt{E_{t}}a^{H}\left(\phi_{0}^{{}^{\prime }},\theta_{0}^{{}^{\prime }}\right), \end{array}$$

respectively.

Similarly, the uplink and downlink channels between the *i*-th UT and satellite can be given as

(5) $$\begin{array}{c} G_{i}=a\left(\phi_{i},\theta_{i}\right)\sqrt{E_{r}}l\left(d\right)\sqrt{G_{t}}b^{H}\left(\omega_{i}^{{}^{\prime }},\upsilon_{i}^{{}^{\prime }}\right) \end{array}$$

and

(6) $$\begin{array}{c} H_{i}=\rho_{i}b\left(\omega_{i},\upsilon_{i}\right)\sqrt{G_{r}}l\left(d\right)\sqrt{E_{t}}a^{H}\left(\phi_{i}^{{}^{\prime }},\theta_{i}^{{}^{\prime }}\right), \end{array}$$

respectively. Here, $\rho_{i}∼\mathrm{CN}\left(0,1\right)$, i∈{0,1,…,M}, denotes the small-scale fading coefficient.

### 2.2. Spectrum Isolation Transmission Method

**Figure 2:**
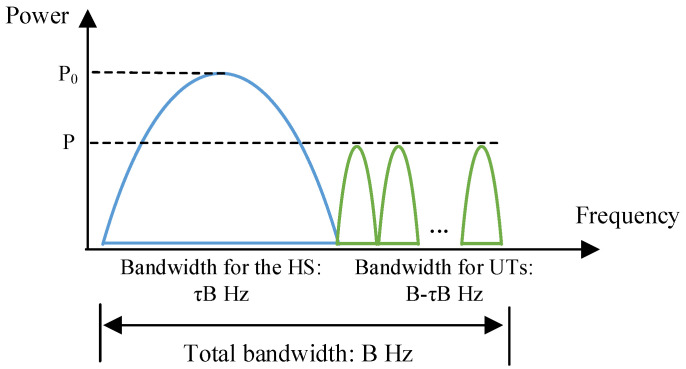
Spectrum allocation in conventional schemes.

To avoid incurring extra interferences, the signals of HS and UTs are designed as spectrum-isolated, which is shown in Figure 2. With a total bandwidth *B* Hz, the HS takes τB Hz, and the rest is for the UTs. Here, 0<τ<1 is the bandwidth factor of the HS.

Let $w_{0,S}$ ($v_{0,S}$) and $w_{U,S}$ ($v_{U,S}$) denote the transmit (receive) beamforming vectors of the feeder and service beams at the satellite, respectively. The transmit and receive beamforming vectors at the *i*-th UT are represented by $w_{i}$ and $v_{i}$, respectively. Since the HS and UTs are allocated separately inside the whole frequency band, broadcasting and information collection could be carried out noninterferingly. The signals arriving at the satellite are given by

(7) $$\begin{array}{cc} \; & x_{0}=v_{0,S}^{H}\left(g_{0}s_{0}+n\right)=v_{0,S}^{H}a\left(\phi_{0},\theta_{0}\right)\sqrt{E_{r}}l\left(d\right)\sqrt{G_{t,0}}s_{0}+v_{0,S}^{H}n,\\ \; & x_{1}=v_{U,S}^{H}\left(G_{1}w_{1}s_{1}+n\right)=v_{U,S}^{H}a\left(\phi_{1},\theta_{1}\right)\sqrt{E_{r}}l\left(d\right)\sqrt{G_{t}}b^{H}\left(\omega_{1}^{{}^{\prime }},\upsilon_{1}^{{}^{\prime }}\right)w_{1}s_{1}+v_{U,S}^{H}n,\\ \; & \cdots ,\\ \; & x_{M}=v_{U,S}^{H}\left(G_{M}w_{M}s_{M}+n\right)=v_{U,S}^{H}a\left(\phi_{M},\theta_{M}\right)\sqrt{E_{r}}l\left(d\right)\sqrt{G_{t}}b^{H}\left(\omega_{M}^{{}^{\prime }},\upsilon_{M}^{{}^{\prime }}\right)w_{M}s_{M}+v_{U,S}^{H}n, \end{array}$$

where $s_{0}$ and $s_{i}$ represent the broadcasting and *i*-th UT signals, respectively. The background noise is denoted as $n∼\mathrm{CN}(0,\sigma^{2}I)$, whose power can be found as $\sigma^{2}$.

Next, the satellite pushes forward the UTs’ signals to the HS and broadcasts the HS’s signals to the UTs. In this case, the *M* signals arriving at the HS can be calculated as

(8) $$\begin{array}{cc} y_{1,0} & =h_{0}^{H}w_{0,S}\left(v_{U,S}^{H}\left(G_{1}w_{1}s_{1}+n\right)\right)+z\\ \; & =\rho_{0}\sqrt{G_{r,0}}l\left(d\right)\sqrt{E_{t}}a^{H}\left(\phi_{0}^{{}^{\prime }},\theta_{0}^{{}^{\prime }}\right)w_{0,S}\left(v_{U,S}^{H}a\left(\phi_{1},\theta_{1}\right)\sqrt{E_{r}}l\left(d\right)\sqrt{G_{t}}b^{H}\left(\omega_{1}^{{}^{\prime }},\upsilon_{1}^{{}^{\prime }}\right)w_{1}s_{1}+v_{U,S}^{H}n\right)+z,\\ \; & \cdots ,\\ y_{M,0} & =h_{0}^{H}w_{0,S}\left(v_{U,S}^{H}\left(G_{M}w_{M}s_{M}+n\right)\right)+z\\ \; & =\rho_{0}\sqrt{G_{r,0}}l\left(d\right)\sqrt{E_{t}}a^{H}\left(\phi_{0}^{{}^{\prime }},\theta_{0}^{{}^{\prime }}\right)w_{0,S}\left(v_{U,S}^{H}a\left(\phi_{M},\theta_{M}\right)\sqrt{E_{r}}l\left(d\right)\sqrt{G_{t}}b^{H}\left(\omega_{M}^{{}^{\prime }},\upsilon_{M}^{{}^{\prime }}\right)w_{M}s_{M}+v_{U,S}^{H}n\right)+z, \end{array}$$

where $z∼\mathrm{CN}(0,\sigma^{2})$ denotes the background noise at the HS.

For the UTs, we have

(9) $$\begin{array}{cc} y_{0,1} & =v_{1}^{H}\left(H_{1}w_{U,S}\left(v_{0,S}^{H}\left(g_{0}s_{0}+n\right)\right)+z\right)\\ \; & =v_{1}^{H}\left(\rho_{1}b\left(\omega_{1},\upsilon_{1}\right)\sqrt{G_{r}}l\left(d\right)\sqrt{E_{t}}a^{H}\left(\phi_{1}^{{}^{\prime }},\theta_{1}^{{}^{\prime }}\right)w_{U,S}\left(v_{0,S}^{H}\left(g_{0}s_{0}+n\right)\right)+z\right),\\ \; & \cdots ,\\ y_{0,M} & =v_{M}^{H}\left(H_{M}w_{U,S}\left(v_{0,S}^{H}\left(g_{0}s_{0}+n\right)\right)+z\right)\\ \; & =v_{M}^{H}\left(\rho_{M}b\left(\omega_{M},\upsilon_{M}\right)\sqrt{G_{r}}l\left(d\right)\sqrt{E_{t}}a^{H}\left(\phi_{M}^{{}^{\prime }},\theta_{M}^{{}^{\prime }}\right)w_{U,S}\left(v_{0,S}^{H}\left(g_{0}s_{0}+n\right)\right)+z\right), \end{array}$$

where $z∼\mathrm{CN}(0,\sigma^{2}I)$ denotes the background noise at UTs.

Considering the computing capability on satellites, beam steering is applied throughout the paper, where ${\left||w|\right|}^{2}={\left||v|\right|}^{2}=1$ [26]. Then, SNRs at the HS can be computed as

(10) $$\begin{array}{cc} \; & SNR_{1,0}=\frac{|h_{0}^{H}w_{0,S}v_{U,S}^{H}G_{1}w_{1}{|}^{2}P_{1}}{\left(|\right|h_{0}^{H}w_{0,S}v_{U,S}^{H}{\left|\right|}^{2}+1)\frac{B-\tau B}{M}\sigma^{2}}=\frac{|h_{0}^{H}w_{0,S}v_{U,S}^{H}G_{1}w_{1}{|}^{2}P}{\left(\right|h_{0}^{H}w_{0,S}{|}^{2}+1)\frac{B-\tau B}{M}\sigma^{2}},\\ \; & \cdots ,\\ \; & SNR_{M,0}=\frac{|h_{0}^{H}w_{0,S}v_{U,S}^{H}G_{M}w_{M}{|}^{2}P_{M}}{\left(|\right|h_{0}^{H}w_{0,S}v_{U,S}^{H}{\left|\right|}^{2}+1)\frac{B-\tau B}{M}\sigma^{2}}=\frac{|h_{0}^{H}w_{0,S}v_{U,S}^{H}G_{M}w_{M}{|}^{2}P}{\left(\right|h_{0}^{H}w_{0,S}{|}^{2}+1)\frac{B-\tau B}{M}\sigma^{2}}, \end{array}$$

where $P_{1}=\ldots =P_{M}=P$ represents the power of the UTs’ signals.

For SNRs at UTs, we have

(11) $$\begin{array}{cc} \; & SNR_{0,1}=\frac{|v_{1}^{H}H_{1}w_{U,S}v_{0,S}^{H}g_{0}{|}^{2}P_{0}}{\left(|\right|v_{1}^{H}H_{1}w_{U,S}v_{0,S}^{H}{\left|\right|}^{2}+\left|\right|v_{1}^{H}{\left|\right|}^{2})\tau B\sigma^{2}}=\frac{|v_{1}^{H}H_{1}w_{U,S}v_{0,S}^{H}g_{0}{|}^{2}P_{0}}{\left(\right|v_{1}^{H}H_{1}w_{U,S}{|}^{2}+1)\tau B\sigma^{2}},\\ \; & \cdots ,\\ \; & SNR_{0,M}=\frac{|v_{M}^{H}H_{M}w_{U,S}v_{0,S}^{H}g_{0}{|}^{2}P_{0}}{\left(|\right|v_{M}^{H}H_{M}w_{U,S}v_{0,S}^{H}{\left|\right|}^{2}+\left|\right|v_{M}^{H}{\left|\right|}^{2})\tau B\sigma^{2}}=\frac{|v_{M}^{H}H_{M}w_{U,S}v_{0,S}^{H}g_{0}{|}^{2}P_{0}}{\left(\right|v_{M}^{H}H_{M}w_{U,S}{|}^{2}+1)\tau B\sigma^{2}}, \end{array}$$

where $P_{0}$ is the signal power of the HS.

Assuming $|v_{1}^{H}H_{1}w_{U,S}{|}^{2}<\ldots <{\left|v_{M}^{H}H_{M}w_{U,S}\right|}^{2}$, we can derive the achievable rate of the HS’s signal as

(12) $$\begin{array}{cc} R_{0} & =\min\{\tau B\log_{2}\left(1+\frac{|v_{1}^{H}H_{1}w_{U,S}v_{0,S}^{H}g_{0}{|}^{2}P_{0}}{\left(\right|v_{1}^{H}H_{1}w_{U,S}{|}^{2}+1)\tau B\sigma^{2}}\right),\ldots ,\tau B\log_{2}\left(1+\frac{|v_{M}^{H}H_{M}w_{U,S}v_{0,S}^{H}g_{0}{|}^{2}P_{0}}{\left(\right|v_{M}^{H}H_{M}w_{U,S}{|}^{2}+1)\tau B\sigma^{2}}\right)\}\\ \; & =\tau B\log_{2}\left(1+\frac{|v_{1}^{H}H_{1}w_{U,S}v_{0,S}^{H}g_{0}{|}^{2}P_{0}}{\left(\right|v_{1}^{H}H_{1}w_{U,S}{|}^{2}+1)\tau B\sigma^{2}}\right). \end{array}$$

Similarly, the achievable rates of the UTs’ signals can be calculated as

(13) $$\begin{array}{cc} \; & R_{1}=\frac{B-\tau B}{M}\log_{2}\left(1+\frac{|h_{0}^{H}w_{0,S}v_{U,S}^{H}G_{1}w_{1}{|}^{2}P}{\left(\right|h_{0}^{H}w_{0,S}{|}^{2}+1)\frac{B-\tau B}{M}\sigma^{2}}\right),\\ \; & \cdots ,\\ \; & R_{M}=\frac{B-\tau B}{M}\log_{2}\left(\frac{|h_{0}^{H}w_{0,S}v_{U,S}^{H}G_{M}w_{M}{|}^{2}P}{\left(\right|h_{0}^{H}w_{0,S}{|}^{2}+1)\frac{B-\tau B}{M}\sigma^{2}}\right). \end{array}$$

## 3. Spectrum-Saving Transmission Scheme

**Figure 3:**
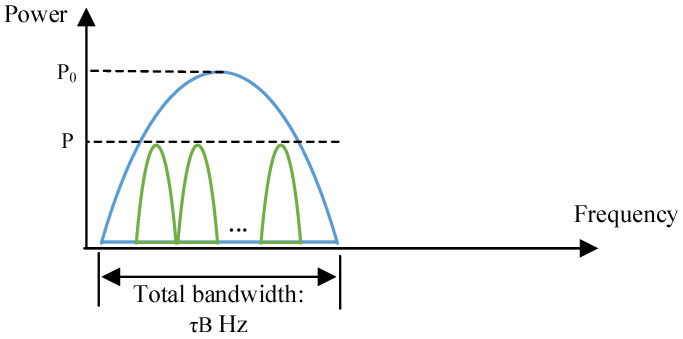
Spectrum allocation in the proposed scheme.

It can be noticed from Section 2.2 that the HS and UTs take spectral resource separately. In this case, more UTs are not allowed to access the network when the spectrum is entirely occupied. Realizing that the receiving capability of the HS is much stronger than that of UTs, we propose a spectrum-saving transmission scheme, which is shown in Figure 3.

In Figure 3, we can observe that UTs are allocated inside the frequency band occupied by the HS. That is, the remaining spectral resource can be saved. In this paper, terminals equipped at users are identical, and transmit the same type of service with different information. That is, all UTs’ signals can be assumed to have the same power level. Under the proposed mechanism, the signal received by the satellite becomes

(14) $$\begin{array}{c} x=v_{0,S}^{H}\left(g_{0}s_{0}+n\right)+v_{U,S}^{H}\left(\sum_{i=1}^{M}G_{i}w_{i}s_{i}+n\right) \end{array}$$

Then, the satellite transmits the co-spectrum signal to the HS and UTs, whose received signals can be expressed as

(15) $$\begin{array}{cc} y_{0} & =h_{0}^{H}w_{0,S}x+z\\ \; & =h_{0}^{H}w_{0,S}\left(v_{0,S}^{H}\left(g_{0}s_{0}+n\right)+v_{U,S}^{H}\left(\sum_{i=1}^{M}G_{i}w_{i}s_{i}+n\right)\right)+z,\\ y_{1} & =v_{1}^{H}\left(H_{1}w_{U,S}x+z\right)\\ \; & =v_{1}^{H}\left(H_{1}w_{U,S}\left(v_{0,S}^{H}\left(g_{0}s_{0}+n\right)+v_{U,S}^{H}\left(\sum_{i=1}^{M}G_{i}w_{i}s_{i}+n\right)\right)+z\right)\\ \; & \cdots ,\\ y_{M} & =v_{M}^{H}\left(H_{M}w_{U,S}x+z\right)\\ \; & =v_{M}^{H}\left(H_{M}w_{U,S}\left(v_{0,S}^{H}\left(g_{0}s_{0}+n\right)+v_{U,S}^{H}\left(\sum_{i=1}^{M}G_{i}w_{i}s_{i}+n\right)\right)+z\right). \end{array}$$

**Figure 4:**
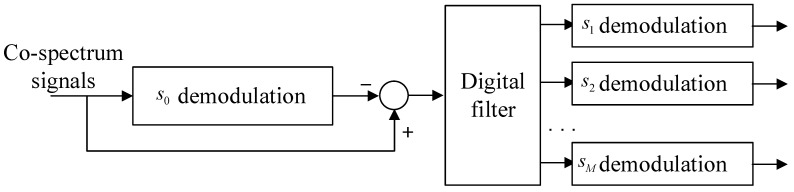
Structure of the receiver at the HS.

To decode the UTs’ signals from the co-spectrum signal *x*, the receiver at the HS should be redesigned, which is shown in Figure 4.

In the first step, the HS demodulates $s_{0}$ directly, where the signal-to-interference-noise ratio (SINR) can be derived as

(16) $$\begin{array}{cc} SINR_{0,0} & =\frac{|h_{0}^{H}w_{0,S}v_{0,S}^{H}g_{0}{|}^{2}P_{0}}{|h_{0}^{H}w_{0,S}{|}^{2}\sum_{i=1}^{M}|v_{U,S}^{H}G_{i}w_{i}{|}^{2}P_{i}+\left(|\right|h_{0}^{H}w_{0,S}v_{0,S}^{H}{\left|\right|}^{2}+\left|\right|h_{0}^{H}w_{0,S}v_{U,S}^{H}{\left|\right|}^{2}+1)\tau B\sigma^{2}}\\ \; & =\frac{|h_{0}^{H}w_{0,S}{|}^{2}{\left|v_{0,S}^{H}g_{0}\right|}^{2}P_{0}}{|h_{0}^{H}w_{0,S}{|}^{2}\sum_{i=1}^{M}|v_{U,S}^{H}G_{i}w_{i}{|}^{2}P+\left(2\right|h_{0}^{H}w_{0,S}{|}^{2}+1)\tau B\sigma^{2}}. \end{array}$$

After canceling $s_{0}$, the *M* signals would be filtered in parallel at each UT’s frequency band. Then, the *M* signals can be derived where the SNRs can be expressed as

(17) $$\begin{array}{cc} \; & SNR_{1,0}=\frac{|h_{0}^{H}w_{0,S}v_{U,S}^{H}G_{1}w_{1}{|}^{2}P_{1}}{\left(|\right|h_{0}^{H}w_{0,S}v_{0,S}^{H}{\left|\right|}^{2}+\left|\right|h_{0}^{H}w_{0,S}v_{U,S}^{H}{\left|\right|}^{2}+1)\frac{\tau B}{M}\sigma^{2}}=\frac{|h_{0}^{H}w_{0,S}{|}^{2}{\left|v_{U,S}^{H}G_{1}w_{1}\right|}^{2}P}{\left(2\right|h_{0}^{H}w_{0,S}{|}^{2}+1)\frac{\tau B}{M}\sigma^{2}},\\ \; & \cdots ,\\ \; & SNR_{M,0}=\frac{|h_{0}^{H}w_{0,S}v_{U,S}^{H}G_{M}w_{M}{|}^{2}P_{M}}{\left(|\right|h_{0}^{H}w_{0,S}v_{0,S}^{H}{\left|\right|}^{2}+\left|\right|h_{0}^{H}w_{0,S}v_{U,S}^{H}{\left|\right|}^{2}+1)\frac{\tau B}{M}\sigma^{2}}=\frac{|h_{0}^{H}w_{0,S}{|}^{2}{\left|v_{U,S}^{H}G_{M}w_{M}\right|}^{2}P}{\left(2\right|h_{0}^{H}w_{0,S}{|}^{2}+1)\frac{\tau B}{M}\sigma^{2}}. \end{array}$$

Since $s_{0}$ is stronger than $s_{1},\ldots ,s_{M}$, the receivers at UTs would demodulate the broadcast signal with SINRs given by

(18) $$\begin{array}{cc} SINR_{0,1}\; & =\frac{|v_{1}^{H}H_{1}w_{U,S}v_{0,S}^{H}g_{0}{|}^{2}P_{0}}{\sum_{i=1}^{M}|v_{1}^{H}H_{1}w_{U,S}v_{U,S}^{H}G_{i}w_{i}{|}^{2}P_{i}+\left(|\right|v_{1}^{H}H_{1}w_{U,S}v_{0,S}^{H}{\left|\right|}^{2}+\left|\right|v_{1}^{H}H_{1}w_{U,S}v_{U,S}^{H}{\left|\right|}^{2}+1)\tau B\sigma^{2}}\\ \; & =\frac{|v_{1}^{H}H_{1}w_{U,S}{|}^{2}{\left|v_{0,S}^{H}g_{0}\right|}^{2}P_{0}}{|v_{1}^{H}H_{1}w_{U,S}{|}^{2}\sum_{i=1}^{M}|v_{U,S}^{H}G_{i}w_{i}{|}^{2}P+\left(2\right|v_{1}^{H}H_{1}w_{U,S}{|}^{2}+1)\tau B\sigma^{2}},\\ \; & \cdots ,\\ SINR_{0,M}\; & =\frac{|v_{M}^{H}H_{M}w_{U,S}v_{0,S}^{H}g_{0}{|}^{2}P_{0}}{\sum_{i=1}^{M}|v_{M}^{H}H_{M}w_{U,S}v_{U,S}^{H}G_{i}w_{i}{|}^{2}P_{i}+\left(|\right|v_{M}^{H}H_{M}w_{U,S}v_{0,S}^{H}{\left|\right|}^{2}+\left|\right|v_{M}^{H}H_{M}w_{U,S}v_{U,S}^{H}{\left|\right|}^{2}+1)\tau B\sigma^{2}}\\ \; & =\frac{|v_{M}^{H}H_{M}w_{U,S}{|}^{2}{\left|v_{0,S}^{H}g_{0}\right|}^{2}P_{0}}{|v_{M}^{H}H_{M}w_{U,S}{|}^{2}\sum_{i=1}^{M}|v_{U,S}^{H}G_{i}w_{i}{|}^{2}P+\left(2\right|v_{M}^{H}H_{M}w_{U,S}{|}^{2}+1)\tau B\sigma^{2}}. \end{array}$$

Assume that $|v_{1}^{H}H_{1}w_{U,S}{|}^{2}<\ldots <{\left|v_{M}^{H}H_{M}w_{U,S}\right|}^{2}$. Then, the achievable rate of the HS’s signal in the spectrum-saving scheme can be derived as

(19) $$\begin{array}{cc} R_{0}^{{}^{\prime }} & =\min\{\tau B\log_{2}\left(1+SINR_{0,0}\right),\tau B\log_{2}\left(1+SINR_{0,1}\right),\ldots ,\tau B\log_{2}\left(1+SINR_{0,M}\right)\}\\ \; & =\tau B\log_{2}\left(1+SINR_{0,1}\right). \end{array}$$

Similarly, the achievable rate of UTs’ signals can be calculated as

(20) $$\begin{array}{cc} \; & R_{1}^{{}^{\prime }}=\frac{\tau B}{M}\log_{2}\left(1+SNR_{1,0}\right),\\ \; & \cdots ,\\ \; & R_{M}^{{}^{\prime }}=\frac{\tau B}{M}\log_{2}\left(1+SNR_{M,0}\right). \end{array}$$

## 4. Performance Evaluation

In this section, we evaluate the performance in terms of ergodic sum rate. Further, the system availability for the proposed method is also discussed. Considering the beamforming style adopted in the paper, we can derive the following assumptions.

**Assumption** **1.** *Since the HS is a central fixed station, the feeder beam of the satellite could be aligned without gain loss. Hence, the uplink and downlink channel gains between the HS and the satellite can be, respectively, written as*

(21) $$\begin{array}{c} |g_{0}\left|\;=\;\right|v_{0,S}^{H}g_{0}|\;=\sqrt{E_{r}}l\left(d\right)\sqrt{G_{t,0}} \end{array}$$

*and*

(22) $$\begin{array}{c} |h_{0}\left|\;=\;\right|h_{0}^{H}w_{0,S}\left|\;=\;\right|\rho_{0}|\sqrt{G_{r,0}}l\left(d\right)\sqrt{E_{t}}. \end{array}$$

Following the principles proposed in [27], we have the following results for the service beam.

**Assumption** **2.** *Since the satellite generates one user beam, and UTs are randomly located in the coverage area, the M beams at UTs could not be all aligned to the service beam. Therefore, we can approximate the service uplink and downlink channel gains as*

(23) $$\begin{array}{c} |g_{i}\left|\;=\;\right|v_{U,S}^{H}G_{i}w_{i}|\;=\sqrt{\eta_{i}E_{r}}l\left(d\right)\sqrt{G_{t}} \end{array}$$

*and*

(24) $$\begin{array}{c} |h_{i}\left|\;=\;\right|v_{i}^{H}H_{i}w_{U,S}\left|\;=\;\right|\rho_{i}|\sqrt{\kappa_{i}G_{r}}l\left(d\right)\sqrt{E_{t}}, \end{array}$$

*where $\eta_{i}\in \left[0.5,1\right]$ and $\kappa_{i}\in \left[0.5,1\right]$ (we set 0.5 as lower bound, as half power beam width is considered for the service beam) denote the beamforming gains of uplink and downlink channels, respectively.*

### 4.1. Ergodic Sum Rate of the Spectrum Isolation Method

Based on the assumptions given above, we study the ergodic sum rate of the conventional spectrum isolation transmission method. As the HS is much stronger than the UTs, the signal power satisfies $P=\alpha P_{0}$, 0<α<1. Define $\beta =P_{0}/\sigma^{2}$ and $E\left[R\right]=E\left[R_{0}\right]+E\left[R_{1}\right]+\ldots +E\left[R_{M}\right]$. The expression of ergodic sum rate is given in the following theorem.

**Theorem** **1.** *The ergodic sum rate of the spectrum isolation method for the SATCOM star network is given by*

(25) $$\begin{array}{cc} E[R] & =E\left[R_{0}\right]+\sum_{i=1}^{M}E\left[R_{i}\right]\\ \; & =\frac{\tau B}{\ln2}\int_{0}^{\frac{|g_{0}{|}^{2}\beta }{\tau B}}\frac{e^{-\frac{1}{L_{1}}\frac{\tau Bx}{|g_{0}{|}^{2}\beta -\tau Bx}}}{1+x}dx+\frac{B-\tau B}{M}\frac{1}{\ln2}\sum_{i=1}^{M}\int_{0}^{\frac{M|g_{i}{|}^{2}\alpha \beta }{\left(B-\tau B\right)}}\frac{e^{-\frac{1}{L_{0}}\frac{(B-\tau B)x}{M|g_{i}{|}^{2}\alpha \beta -\left(B-\tau B\right)x}}}{1+x}dx, \end{array}$$

*where $L_{1}=\kappa_{1}G_{r}l^{2}\left(d\right)E_{t}$ and $L_{0}=G_{r,0}l^{2}\left(d\right)E_{t}$.*

**Proof.**  According to (12) and (13), the ergodic sum rate can be expressed as

(26) $$\begin{array}{cc} E[R] & =E\left[R_{0}\right]+\sum_{i=1}^{M}E\left[R_{i}\right]\\ \; & =\tau BE\left[\log_{2}\left(1+\frac{|v_{1}^{H}H_{1}w_{U,S}v_{0,S}^{H}g_{0}{|}^{2}P_{0}}{\left(\right|v_{1}^{H}H_{1}w_{U,S}{|}^{2}+1)\tau B\sigma^{2}}\right)\right]+\sum_{i=1}^{M}\frac{B-\tau B}{M}E\left[\log_{2}\left(1+\frac{|h_{0}^{H}w_{0,S}v_{U,S}^{H}G_{i}w_{i}{|}^{2}P}{\left(\right|h_{0}^{H}w_{0,S}{|}^{2}+1)\frac{B-\tau B}{M}\sigma^{2}}\right)\right]. \end{array}$$

With Assumptions 1 and 2, the above equation can be further calculated as

(27) $$\begin{array}{cc} E[R] & =\tau BE\left[\log_{2}\left(1+\frac{|h_{1}{|}^{2}{\left|g_{0}\right|}^{2}P_{0}}{\left(\right|h_{1}{|}^{2}+1)\tau B\sigma^{2}}\right)\right]+\sum_{i=1}^{M}\frac{B-\tau B}{M}E\left[\log_{2}\left(1+\frac{|h_{0}{|}^{2}{\left|g_{i}\right|}^{2}P}{\left(\right|h_{0}{|}^{2}+1)\frac{B-\tau B}{M}\sigma^{2}}\right)\right]. \end{array}$$

Since $\rho_{i}∼\mathrm{CN}\left(0,1\right)$, i∈{0,1,…,M}, we have $h_{1}∼\mathrm{CN}\left(0,L_{1}\right)$ and $h_{0}∼\mathrm{CN}\left(0,L_{0}\right)$, where $L_{1}=\kappa_{1}G_{r}l^{2}\left(d\right)E_{t}$ and $L_{0}=G_{r,0}l^{2}\left(d\right)E_{t}$. Then, $|h_{1}{|}^{2}∼\exp\left(1/L_{1}\right)$ and $|h_{0}{|}^{2}∼\exp\left(1/L_{0}\right)$ can be obtained. According to the process adopted in [28], the ergodic rate of the HS signal can be written as

(28) $$\begin{array}{c} E\left[R_{0}\right]=\tau BE\left[\log_{2}\left(1+\frac{|h_{1}{|}^{2}{\left|g_{0}\right|}^{2}P_{0}}{\left(\right|h_{1}{|}^{2}+1)\tau B\sigma^{2}}\right)\right]=\frac{\tau B}{\ln2}\int_{0}^{\infty }\frac{1-F_{X}\left(x\right)}{1+x}dx, \end{array}$$

where $X=\frac{|h_{1}{|}^{2}{\left|g_{0}\right|}^{2}P_{0}}{\left(\right|h_{1}{|}^{2}+1)\tau B\sigma^{2}}$.

As $F_{X}\left(x\right)=\mathrm{Pr}\left(\frac{|h_{1}{|}^{2}{\left|g_{0}\right|}^{2}P_{0}}{\left(\right|h_{1}{|}^{2}+1)\tau B\sigma^{2}}\leq x\right)$, we have

(29) $$\begin{array}{c} F_{X}\left(x\right)=\mathrm{Pr}\left(\frac{|h_{1}{|}^{2}{\left|g_{0}\right|}^{2}P_{0}}{\left(\right|h_{1}{|}^{2}+1)\tau B\sigma^{2}}\leq x\right)=\mathrm{Pr}(|h_{1}{|}^{2}\left(1-\frac{\tau B\sigma^{2}x}{|g_{0}{|}^{2}P_{0}}\right)\leq \frac{\tau B\sigma^{2}x}{|g_{0}{|}^{2}P_{0}}). \end{array}$$

In case of $1-\frac{\tau B\sigma^{2}x}{|g_{0}{|}^{2}P_{0}}>0$, i.e., $x<\frac{|g_{0}{|}^{2}P_{0}}{\tau B\sigma^{2}}$, (29) can be calculated as

(30) $$\begin{array}{c} F_{X}\left(x\right)=\mathrm{Pr}(|h_{1}{|}^{2}\leq \frac{\tau B\sigma^{2}x}{|g_{0}{|}^{2}P_{0}-\tau B\sigma^{2}x})=1-e^{-\frac{1}{L_{1}}\frac{\tau B\sigma^{2}x}{|g_{0}{|}^{2}P_{0}-\tau B\sigma^{2}x}} \end{array}$$

When $1-\frac{\tau B\sigma^{2}x}{|g_{0}{|}^{2}P_{0}}<0$, $|h_{1}{|}^{2}\left(1-\frac{\tau B\sigma^{2}x}{|g_{0}{|}^{2}P_{0}}\right)\leq \frac{\tau B\sigma^{2}x}{|g_{0}{|}^{2}P_{0}}$ always holds, i.e., $F_{X}\left(x\right)=1$. Then, we have

(31) $$\begin{array}{c} E\left[R_{0}\right]=\frac{\tau B}{\ln2}\int_{0}^{\infty }\frac{1-F_{X}\left(x\right)}{1+x}dx=\frac{\tau B}{\ln2}\int_{0}^{\frac{|g_{0}{|}^{2}P_{0}}{\tau B\sigma^{2}}}\frac{e^{-\frac{1}{L_{1}}\frac{\tau B\sigma^{2}x}{|g_{0}{|}^{2}P_{0}-\tau B\sigma^{2}x}}}{1+x}dx=\frac{\tau B}{\ln2}\int_{0}^{\frac{|g_{0}{|}^{2}\beta }{\tau B}}\frac{e^{-\frac{1}{L_{1}}\frac{\tau Bx}{|g_{0}{|}^{2}\beta -\tau Bx}}}{1+x}dx. \end{array}$$

Similarly, the ergodic rate of UT *i* can be derived as

(32) $$\begin{array}{c} E\left[R_{i}\right]=\frac{B-\tau B}{M\ln2}\int_{0}^{\frac{M|g_{i}{|}^{2}\alpha \beta }{\left(B-\tau B\right)}}\frac{e^{-\frac{1}{L_{0}}\frac{(B-\tau B)x}{M|g_{i}{|}^{2}\alpha \beta -\left(B-\tau B\right)x}}}{1+x}dx. \end{array}$$

Consequently, (25) can be obtained. The theorem is proved. □

### 4.2. Ergodic Sum Rate of the Proposed Method

For the ergodic sum rate of the spectrum-saving method, we give the following theorem.

**Theorem** **2.** *The ergodic sum rate of the spectrum-saving method for the SATCOM star network is given by*

(33) $$\begin{array}{cc} E[R^{{}^{\prime }}] & =E\left[R_{0}^{{}^{\prime }}\right]+\sum_{i=1}^{M}E\left[R_{i}^{{}^{\prime }}\right]\\ \; & =\frac{\tau B}{\ln2}\int_{0}^{\frac{|g_{0}{|}^{2}\beta }{G\alpha \beta +2\tau B}}\frac{e^{-\frac{1}{L_{1}}\frac{\tau By}{|g_{0}{|}^{2}\beta -G\alpha \beta y-2\tau By}}}{1+y}dy+\frac{\tau B}{M\ln2}\sum_{i=1}^{M}\int_{0}^{\frac{M|g_{i}{|}^{2}\alpha \beta }{2\tau B}}\frac{e^{-\frac{1}{L_{0}}\frac{\tau By}{M|g_{i}{|}^{2}\alpha \beta -2\tau By}}}{1+y}dy, \end{array}$$

*where $G=|g_{1}{|}^{2}+\ldots +{\left|g_{M}\right|}^{2}$.*

**Proof.**  Based on the results derived in Section 3, the ergodic sum rate can be obtained as

(34) $$\begin{array}{cc} \; & E\left[R^{{}^{\prime }}\right]=E\left[R_{0}^{{}^{\prime }}\right]+\sum_{i=1}^{M}E\left[R_{i}^{{}^{\prime }}\right]\\ \; & =\tau BE[\log_{2}\left(1+\frac{|v_{1}^{H}H_{1}w_{U,S}{|}^{2}{\left|v_{0,S}^{H}g_{0}\right|}^{2}P_{0}}{|v_{1}^{H}H_{1}w_{U,S}{|}^{2}\sum_{i=1}^{M}|v_{U,S}^{H}G_{i}w_{i}{|}^{2}P+\left(2\right|v_{1}^{H}H_{1}w_{U,S}{|}^{2}+1)\tau B\sigma^{2}}\right)]\\ \; & +\frac{\tau B}{M}\sum_{i=1}^{M}E\left[\log_{2}\left(1+\frac{|h_{0}^{H}w_{0,S}{|}^{2}{\left|v_{U,S}^{H}G_{i}w_{i}\right|}^{2}P}{\left(2\right|h_{0}^{H}w_{0,S}{|}^{2}+1)\frac{\tau B}{M}\sigma^{2}}\right)\right] \end{array}$$

According to the assumptions, (34) can be further expressed as

(35) $$\begin{array}{c} E\left[R^{{}^{\prime }}\right]=\tau BE\left[\log_{2}\left(1+\frac{|h_{1}{|}^{2}{\left|g_{0}\right|}^{2}P_{0}}{|h_{1}{|}^{2}\sum_{i=1}^{M}|g_{i}{|}^{2}P+\left(2\right|h_{1}{|}^{2}+1)\tau B\sigma^{2}}\right)\right]+\frac{\tau B}{M}\sum_{i=1}^{M}E\left[\log_{2}\left(1+\frac{M|h_{0}{|}^{2}{\left|g_{i}\right|}^{2}P}{\left(2\right|h_{0}{|}^{2}+1)\tau B\sigma^{2}}\right)\right]. \end{array}$$

Let $G=\sum_{i=1}^{M}{\left|g_{i}\right|}^{2}$. Then, we have

(36) $$\begin{array}{c} E\left[R_{0}^{{}^{\prime }}\right]=\tau BE\left[\log_{2}\left(1+\frac{|h_{1}{|}^{2}{\left|g_{0}\right|}^{2}P_{0}}{|h_{1}{|}^{2}GP+\left(2\right|h_{1}{|}^{2}+1)\tau B\sigma^{2}}\right)\right]=\frac{\tau B}{\ln2}\int_{0}^{\infty }\frac{1-F_{Y}\left(y\right)}{1+y}dy, \end{array}$$

where $Y=\frac{|h_{1}{|}^{2}{\left|g_{0}\right|}^{2}P_{0}}{|h_{1}{|}^{2}GP+\left(2\right|h_{1}{|}^{2}+1)\tau B\sigma^{2}}$.

As analyzed in Theorem 1, we have

(37) $$\begin{array}{c} F_{Y}\left(y\right)=\mathrm{Pr}\left(\frac{|h_{1}{|}^{2}{\left|g_{0}\right|}^{2}P_{0}}{|h_{1}{|}^{2}GP+\left(2\right|h_{1}{|}^{2}+1)\tau B\sigma^{2}}\leq y\right)=\mathrm{Pr}(|h_{1}{|}^{2}\left(\right|g_{0}{|}^{2}P_{0}-GPy-2\tau B\sigma^{2}y)\leq \tau B\sigma^{2}y). \end{array}$$

When $|g_{0}{|}^{2}P_{0}-GPy-2\tau B\sigma^{2}y>0$, i.e., $y<\frac{|g_{0}{|}^{2}P_{0}}{GP+2\tau B\sigma^{2}}$, the above equation becomes

(38) $$\begin{array}{c} F_{Y}\left(y\right)=\mathrm{Pr}(|h_{1}{|}^{2}\leq \frac{\tau B\sigma^{2}y}{|g_{0}{|}^{2}P_{0}-GPy-2\tau B\sigma^{2}y})=1-e^{-\frac{1}{L_{1}}\frac{\tau B\sigma^{2}y}{|g_{0}{|}^{2}P_{0}-GPy-2\tau B\sigma^{2}y}} \end{array}$$

Otherwise, $F_{Y}\left(y\right)=1$. Then, (36) can be expressed as

(39) $$\begin{array}{c} E\left[R_{0}^{{}^{\prime }}\right]=\frac{\tau B}{\ln2}\int_{0}^{\frac{|g_{0}{|}^{2}P_{0}}{GP+2\tau B\sigma^{2}}}\frac{e^{-\frac{1}{L_{1}}\frac{\tau B\sigma^{2}y}{|g_{0}{|}^{2}P_{0}-GPy-2\tau B\sigma^{2}y}}}{1+y}dy=\frac{\tau B}{\ln2}\int_{0}^{\frac{|g_{0}{|}^{2}\beta }{G\alpha \beta +2\tau B}}\frac{e^{-\frac{1}{L_{1}}\frac{\tau By}{|g_{0}{|}^{2}\beta -G\alpha \beta y-2\tau By}}}{1+y}dy. \end{array}$$

Following the same process, the ergodic rate of UT *i* can be calculated as

(40) $$\begin{array}{c} E\left[R_{i}^{{}^{\prime }}\right]=\frac{\tau B}{M\ln2}\int_{0}^{\frac{M|g_{i}{|}^{2}\alpha \beta }{2\tau B}}\frac{e^{-\frac{1}{L_{0}}\frac{\tau By}{M|g_{i}{|}^{2}\alpha \beta -2\tau By}}}{1+y}dy. \end{array}$$

With (39) and (40), we can derive (33). The theorem is proved. □

### 4.3. Impacts of UTs on System Availability

As a UT is designed to demodulate the signal of the HS with the interference of all UT signals, BER performance should be guaranteed when using the spectrum-saving scheme. To give instructions on the proposed method, we provide the definition of system availability as follows.

**Definition** **1.** *Assume that UT 1 has the worst channel condition. With a given threshold Γ, the system availability of the spectrum-saving scheme is defined as $P_{ava}=P\left(SINR_{0,1}\geq \Gamma \right)$.*

In this case, the system availability can be calculated as

(41) $$\begin{array}{cc} P_{ava} & =P(SINR_{0,1}\geq \Gamma )\\ \; & =P(\frac{|h_{1}{|}^{2}{\left|g_{0}\right|}^{2}\beta }{|h_{1}{|}^{2}G\alpha \beta +\left(2\right|h_{1}{|}^{2}+1)\tau B}\geq \Gamma )\\ \; & =1-P(\frac{|h_{1}{|}^{2}{\left|g_{0}\right|}^{2}\beta }{|h_{1}{|}^{2}G\alpha \beta +\left(2\right|h_{1}{|}^{2}+1)\tau B}<\Gamma )\\ \; & =e^{-\frac{1}{L_{1}}\frac{\tau B\Gamma }{|g_{0}{|}^{2}\beta -G\alpha \beta \Gamma -2\tau B\Gamma }}. \end{array}$$

We can find that the system availability degrades with the number of UTs.

## 5. Simulation Results

In this section, we provide simulation results to validate the performance superiority of the spectrum-saving transmission method. The system is assumed to be working in Ku band with B=1 Hz where $\lambda =c/f=3\times 10^{8}/\left(14\times 10^{9}\right)$ m. In this paper, we consider a geostationary Earth orbit satellite where the orbit height is set to *d* = 36,000 km. Moreover, the normal-direction gains of transmit and receive beams of the satellite are set to $E_{t}=120$ dB and $E_{r}=50$ dB, respectively. Considering the strong capability of the HS, we set $G_{t,0}=82$ dB and $G_{r,0}=61$ dB, while the normal-direction gains of UT UPAs are set to $G_{t}=45$ dB and $G_{r}=37$ dB. In addition, $\eta_{i}$ and $\kappa_{i}$ are randomly generated in [0.5,1].

### 5.1. Ergodic Sum Rate

**Figure 5:**
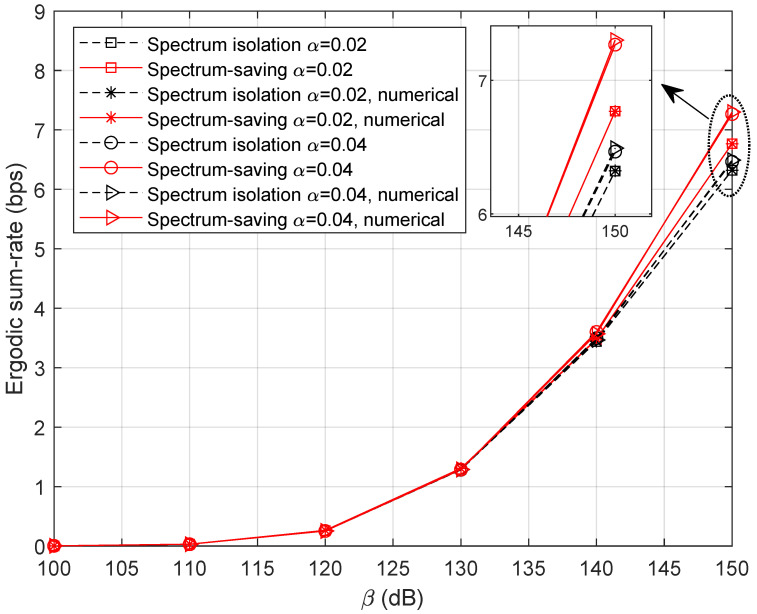
Ergodic sum rate with variable β when M=5.

The signal power of the HS is assumed to be $P_{0}=1$ W. Letting M=5, τ=0.8, and α={0.02,0.04}, simulation results of the ergodic sum rate versus β are shown in Figure 5. As expected, the curves of theoretical expression perfectly match with those of numerical results, which validates the effectiveness of Theorems 1 and 2. By deeply analyzing the curves, we can clearly find that the spectrum-saving scheme outperforms the conventional method in the high-SNR region. This can be explained as the benefit of extended bandwidth allocated for UTs is much more noticeable than the interference incurred by the new method.

**Figure 6:**
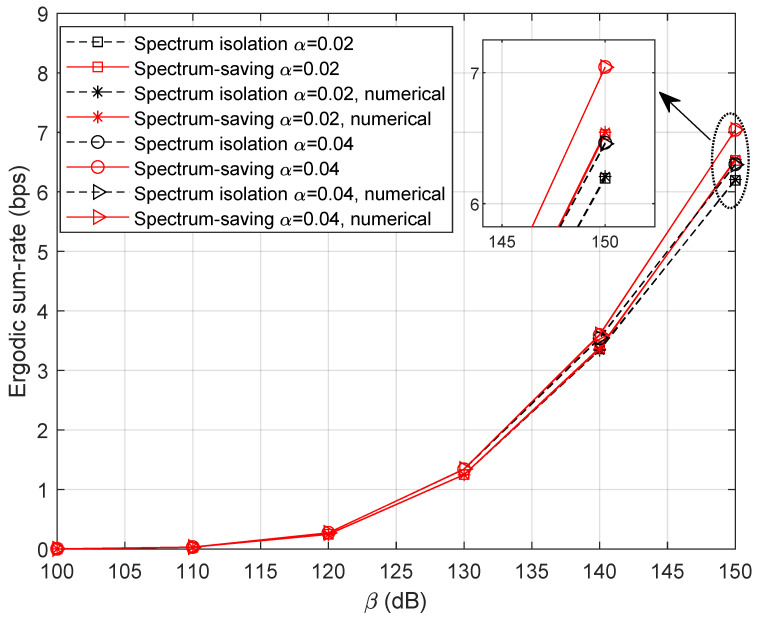
Ergodic sum rate with variable β when M=3.

Figure 6 illustrates the ergodic sum-rate with fewer accessed UTs. Here, we set M=3. It can be observed that the ergodic sum rate decreases compared with the results in Figure 5. In addition, the spectrum-saving approach still outperforms the conventional method.

**Figure 7:**
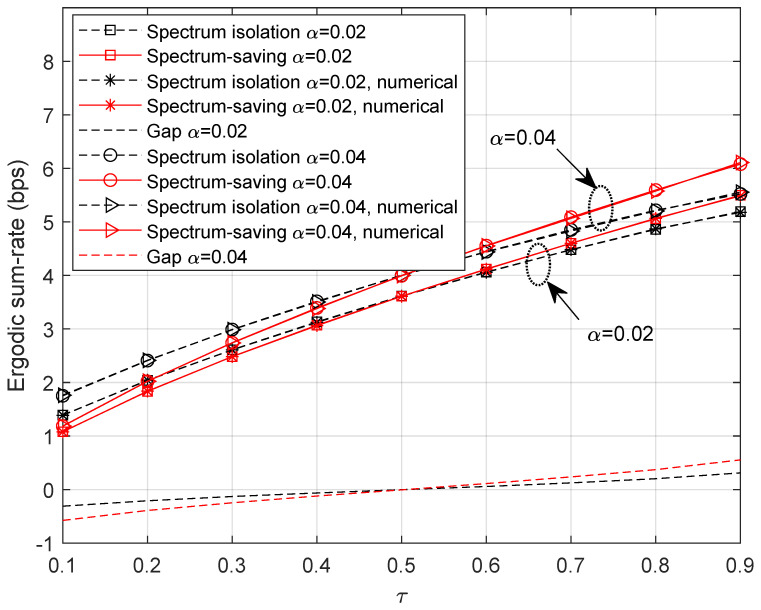
Ergodic sum rate with variable τ.

To learn more about the effect of shared bandwidth on the ergodic sum rate, we ran simulations with different values of τ, where the result is presented in Figure 7. Specifically, β is set to 145 dB and α={0.02,0.04} in this scenario. Obviously, more competitive results can be obtained by increasing the power factor α for UTs’ signals. It is noteworthy that the proposed method is not satisfactory when τ is relatively small. However, the performance gain becomes noticeable as the bandwidth grows, which can be learned from the ‘Gap’ curve. In this sense, we can expect promising performances by designing a proper value of τ.

### 5.2. BER Performance

**Figure 8:**
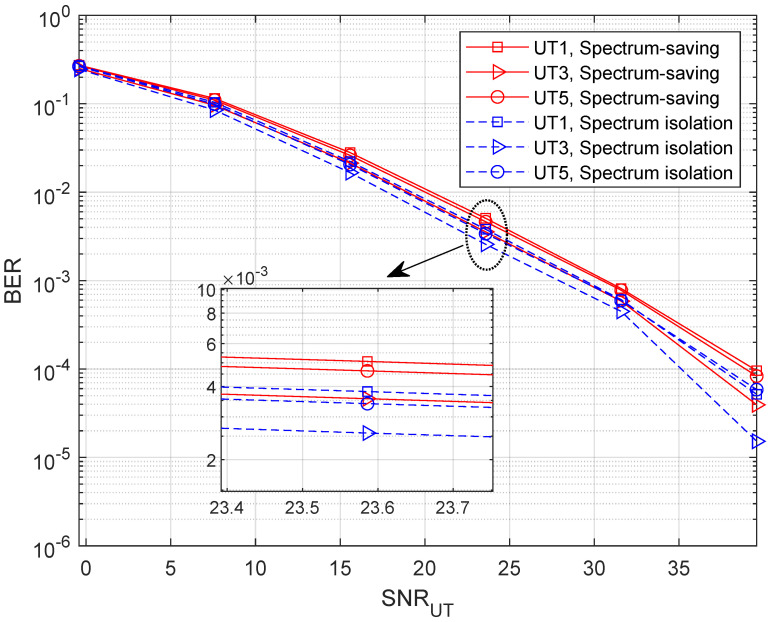
BER with variable SNR${}_{\mathrm{UT}}$ when M=5.

**Figure 9:**
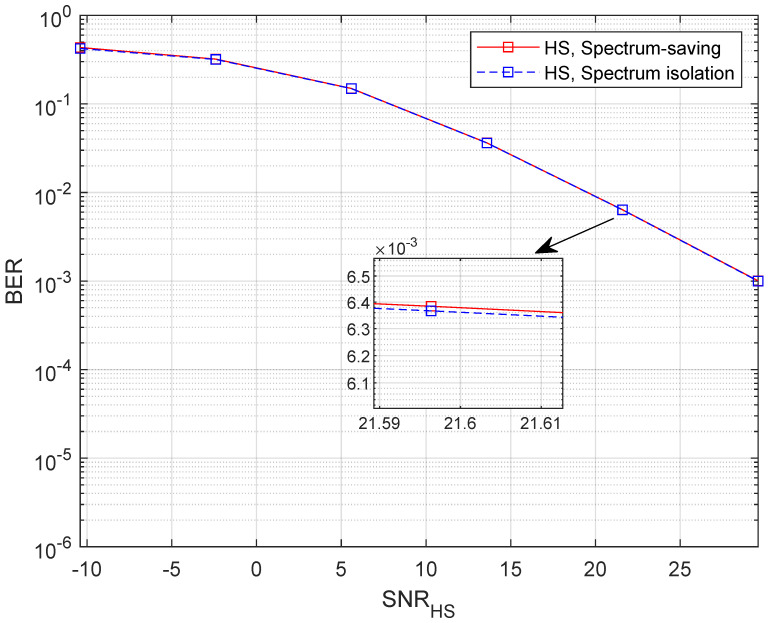
BER with variable SNR${}_{\mathrm{HS}}$ when M=5.

To find out how the receiver behaves in the spectrum-saving scheme, we provide BER results in this subsection, where M=5. Note that we only consider the unaligned beamforming degradation factors and receiving capability differences in building the HS and UT channel gains. Specifically, 4-QAM with gray mapping is applied in simulations where the bit length is $1024\times 10^{4}$. Compared with UTs, the receiving capability advantage of the HS is 7 dB. We define the link SNR at UTs as $\left(P_{0}+M\times P\right)/\sigma^{2}$, and the *x*-axis values are presented as $SNR_{UT}=P_{0}/\sigma^{2}$ and $SNR_{HS}=P/\sigma^{2}$ for UT and HS receivers, respectively. To show the results clearly, we present BER curves of three UTs instead of all the UTs. It can be understood from Figure 8 that the BER slightly degrades when applying the spectrum-saving scheme since signals of other UTs are regarded as interferences. Further, we can find from Figure 9 that BER performance at the HS is comparable to the spectrum isolation method since SIC and parallel filtering is considered.

### 5.3. Impact of *M*

**Figure 10:**
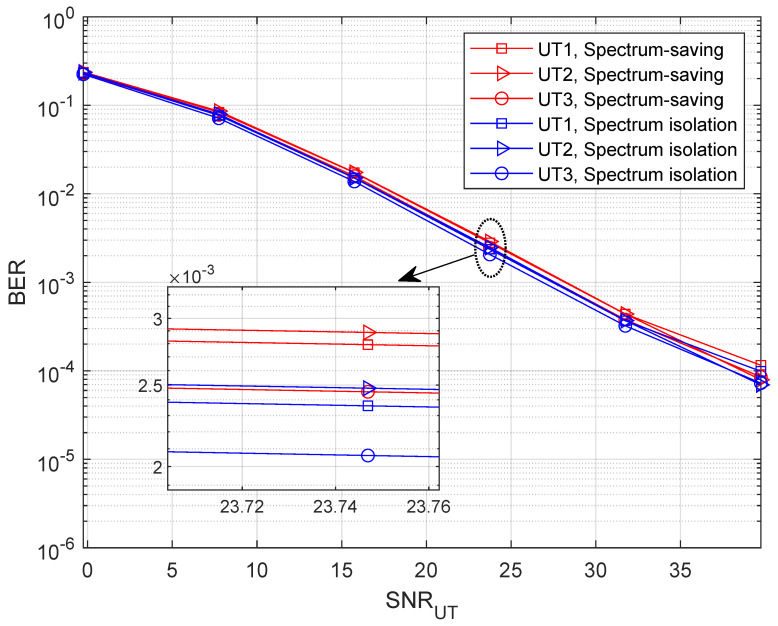
BER with variable SNR${}_{\mathrm{UT}}$ when M=3.

As analyzed in Section 4.3, system availability is affected by the number of accessed UTs. In Figure 10, we provide the BER performance with M=3. It can be clearly noticed that the performance degradation of the proposed method is marginal at UTs compared with the spectrum isolation scheme. In addition, the BER results are more promising than those in Figure 8. Consequently, we can derive satisfactory results by adjusting the number of accessed UTs.

### 5.4. Performance Comparison with MF-TDMA

To adequately verify the advancement of the proposed method, we provide simulation results of MF-TDMA [29] as another benchmark. Let *N* denote the number of total carriers shared by the *M* UTs. The bandwidth for each carrier can be derived as (B−τB)/N, and the effective time-slot length for each UT is N/M s in unit time. Then, the ergodic sum rate for the MF-TDMA scheme can be derived as

(42) $$\begin{array}{cc} E[\tilde{R}] & =E\left[\tilde{R_{0}}\right]+\sum_{i=1}^{M}E\left[\tilde{R_{i}}\right]\\ \; & =\frac{\tau B}{\ln2}\int_{0}^{\frac{|g_{0}{|}^{2}\beta }{\tau B}}\frac{e^{-\frac{1}{L_{1}}\frac{\tau Bx}{|g_{0}{|}^{2}\beta -\tau Bx}}}{1+x}dx+\frac{N}{M}\frac{B-\tau B}{N}\frac{1}{\ln2}\sum_{i=1}^{M}\int_{0}^{\frac{N|g_{i}{|}^{2}\alpha \beta }{\left(B-\tau B\right)}}\frac{e^{-\frac{1}{L_{0}}\frac{(B-\tau B)x}{N|g_{i}{|}^{2}\alpha \beta -\left(B-\tau B\right)x}}}{1+x}dx. \end{array}$$

**Figure 11:**
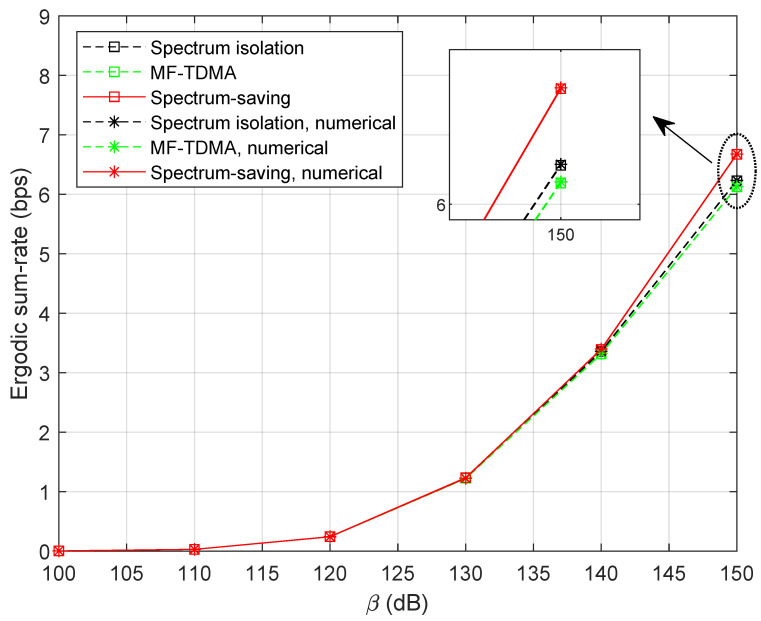
Ergodic sum rate with variable β when M=5 and N=3.

Using the same system parameters as Section 5.1, we provide simulation results of the ergodic sum rate for the three methods in Figure 11, where α=0.02 and N=3. It is noteworthy that the proposed spectrum-saving method can provide a promising performance compared with spectrum isolation and MF-TDMA schemes.

**Figure 12:**
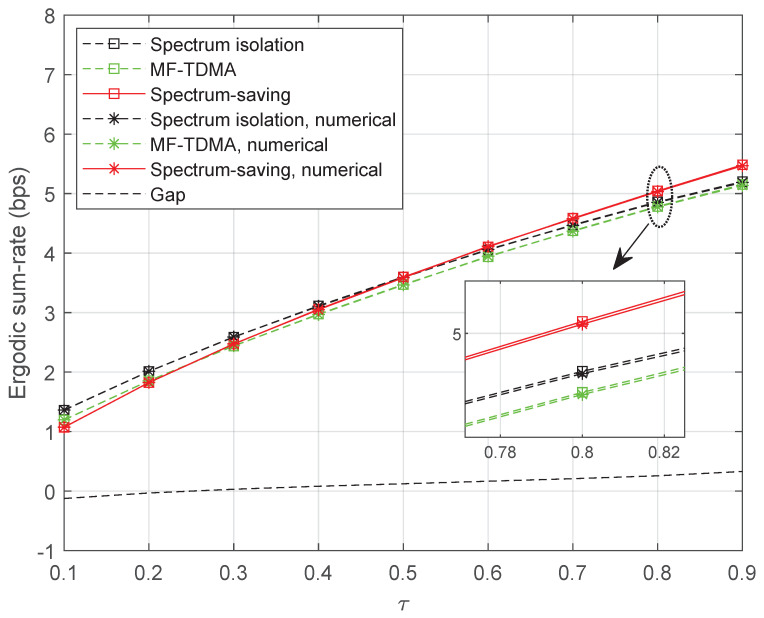
Ergodic sum rate with variable τ when M=5 and N=3.

To discover how the performance gap behaves with shared bandwidth, we provide simulation results of the three methods in Figure 12. Note that the ‘Gap’ curve represents the performance gap between the spectrum-saving and the MF-TDMA schemes. It can be noticed that MF-TDMA is competitive when τ<0.3; however, the spectrum-saving outperforms the other two methods with the increase of shared bandwidth.

## 6. Conclusions

In this paper, we developed a spectrum-saving transmission method in multi-antenna satellite communication systems where beam steering is used at the satellite and UTs. By realizing the different transmission capabilities at the HS and UTs, the spectral resource of the HS was shared with UTs. To successfully demodulate the information of UTs, the SIC and parallel filtering are taken into consideration at the HS. As a further advance, we derived the ergodic sum rate for performance analysis and analyzed the impact of the accessed UTs in terms of the system availability. Simulation results verified that the spectrum-saving method is capable of providing better performances with less spectrum expense, while the BER would not be obviously degraded.

## Data Availability

Not applicable.

## References

[1] Zhang X., Zhu L., Li T., Xia Y., Zhuang W. (2019). Multiple-User transmission in space information networks: Architecture and key techniques. IEEE Wirel. Commun..

[2] Bai L., Zhu L., Zhang X., Zhang W., Quan Y. (2018). Multi-Satellite relay transmission in 5G: Concepts, techniques, and challenges. IEEE Netw..

[3] Kim J., Lee J., Ko H., Kim T., Pack S. (2022). Space mobile networks: Satellite as core and access networks for B5G. IEEE Commun. Mag..

[4] Cao H., Zhu W., Feng W., Fan J. (2022). Robust beamforming based on graph attention networks for IRS-assisted satellite IoT communications. Entropy.

[5] Chen S., Sun S., Kang S. (2020). System integration of terrestrial mobile communication and satellite communication-the trends, challenges and key technologies in B5G and 6G. China Commun..

[6] 3rd Generation Partnership Project (3GPP) (2022). Study on Integration of Satellite Components in the 5G Architecture: TR 23.700-28 v0.3.0. https://www.5g-mobix.com/assets/files/5G-MOBIX-D6.7-Final-report-on-the-standardisation-and-spectrum-allocation-needs-v1.0.pdf.

[7] Sun C. (2017). Research status and problems for space-based transmission network and space-ground integrated information network. Radio Eng..

[8] Li X., Wang H., Zhao W., Tian Q., Xu Z., Yang B. (2022). A new multiple gateway transmit diversity technique for future satellite networks. China Commun..

[9] Rana A.H., McCoskey J., Check W. (1990). VSAT technology, trends, and applications. Proc. IEEE.

[10] Ma Y., Jiang H., Li J., Yu H., Li C., Zhang D. Design of marine satellite communication system based on VSAT technique. Proceedings of the 2021 International Conference on CITCE.

[11] Lacoste C., Martins W., Chatzinotas S., Emiliani L. (2022). Inbound carrier plan optimization for adaptive VSAT networks. IEEE Trans. Aerosp. Electron. Syst..

[12] Mubarak R., Budiyanto S., Alaydrus M., Adriansyah A. The utilisation of information systems for VSAT development in rural areas. Proceedings of the 2020 2nd International Conference on BCWSP.

[13] Al-Wakeel S., Al-Wakeel M. An architecture design of a VSAT satellite network for multimedia on demand services. Proceedings of the 2000 IEEE WCNC.

[14] Hadjitheodosiou M., Coakley F., Evans B. (1997). Next generation multiservice VSAT networks. Electron. Commun. Eng. J..

[15] Bugaj M. Veryfication of multi-access techniques for VSAT satellite terminals. Proceedings of the 2018 PIERS-Toyama.

[16] Zhu X., Jiang C., Yin L., Kuang L., Ge N., Lu J. (2017). Non-orthogonal multiple access based integrated terrestrial-satellite networks. IEEE J. Sel. Areas Commun..

[17] Zhu X., Jiang C., Yin L., Kuang L., Ge N., Lu J. (2018). Cooperative multigroup multicast transmission in integrated terrestrial-satellite networks. IEEE J. Sel. Areas Commun..

[18] Yan X., Xiao H., An K., Zheng G., Tao W. (2018). Hybrid satellite terrestrial relay networks with cooperative non-orthogonal multiple access. IEEE Commun. Lett..

[19] Yue X., Liu Y., Yao Y., Li T., Li X., Liu R., Nallanathan A. (2020). Outage behaviors of NOMA-based satellite network over Shadowed-Rician fading channels. IEEE Trans. Veh. Technol..

[20] Li T., Hao X., Yue X. (2020). A power domain multiplexing based co-carrier transmission method in hybrid satellite communication networks. IEEE Access.

[21] He G., Gao X., Sun L., Zhang R. (2021). A review of multibeam phased array antennas as LEO satellite constellation ground station. IEEE Access.

[22] Vázquez M.Á., Blanco L., Pérez-Neira A.I. (2018). Spectrum sharing backhaul satellite-terrestrial systems via analog beamforming. IEEE J. Sel. Top. Signal Process..

[23] Moon S., Yun S., Yom I., Lee H. (2019). Phased array shaped-beam satellite antenna with boosted-beam control. IEEE Trans. Antennas Propag..

[24] Alves H., Riihonen T., Suraweera H.A. (2020). Full-Duplex Communications for Future Wireless Networks.

[25] Maral G., Bousquet M., Sun Z. (2020). Satellite Communications Systems: Systems, Techniques and Technology.

[26] Liang X., Jiao J., Wu S., Zhang Q. (2018). Outage analysis of multirelay multiuser hybrid satellite-terrestrial millimeter-wave networks. IEEE Wirel. Commun. Lett..

[27] Bai L., Li T., Xiao Z., Choi J. (2017). Performance analysis for SDMA mmWave systems: Using an approximate closed-form solution of downlink sum-rate. IEEE Access.

[28] Suraweera H.A., Smith P.J., Shafi M. (2010). Capacity limits and performance analysis of cognitive radio with imperfect channel knowledge. IEEE Trans. Veh. Technol..

[29] He Y., Liu Y., Jiang C., Zhong X. (2022). Multiobjective anti-collision for massive access ranging in MF-TDMA satellite communication system. IEEE Internet Things J..

